# The Effects of Alda-1 Treatment on Renal and Intestinal Injuries After Cardiopulmonary Resuscitation in Pigs

**DOI:** 10.3389/fmed.2022.892472

**Published:** 2022-05-12

**Authors:** Qian Yu, Jianbo Gao, Xuebo Shao, Wei Lu, Linling Chen, Lili Jin

**Affiliations:** ^1^Department of Emergency Medicine, The First People's Hospital of Fuyang Hangzhou, Hangzhou, China; ^2^Department of Intensive Care Unit, The First People's Hospital of Fuyang Hangzhou, Hangzhou, China; ^3^Health Commission of Fuyang Hangzhou, Hangzhou, China

**Keywords:** Alda-1, apoptosis, cardiac arrest, cardiopulmonary resuscitation, ferroptosis, intestinal injury, renal injury

## Abstract

**Aim:**

After successful cardiopulmonary resuscitation (CPR), most survivors will develop acute kidney injury and intestinal barrier dysfunction, both of which contribute to the poor outcomes of cardiac arrest (CA) victims. Recently, the aldehyde dehydrogenase 2 (ALDH2) agonist, Alda-1 was shown to effectively alleviate regional ischemia/reperfusion injury of various organs. In the present study, we investigated the effects of Alda-1 treatment on renal and intestinal injuries after CA and resuscitation in pigs.

**Methods:**

Twenty-four male domestic pigs were randomly divided into one of the three groups: sham (*n* = 6), CPR (*n* = 10), or CPR+Alda-1 (*n* = 8). CA was induced and untreated for 8 min, and then CPR was performed for 8 min in the CPR and CPR+Alda-1 groups. At 5 min after resuscitation, a dose of 0.88 mg/kg of Alda-1 was intravenously administered in the CPR+Alda-1 group. The biomarkers of renal and intestinal injuries after resuscitation were regularly measured for a total of 24 h. Subsequently, the animals were euthanized, and then renal and intestinal tissues were obtained for the measurements of ALDH2 activity and expression, and cell apoptosis and ferroptosis.

**Results:**

Five of the 10 animals in the CPR group and six of the eight animals in the CPR+Alda-1 group were successfully resuscitated. After resuscitation, the levels of biomarkers of renal and intestinal injuries were significantly increased in all animals experiencing CA and resuscitation compared with the sham group; however, Alda-1 treatment significantly alleviated renal and intestinal injuries compared to the CPR group. Post-resuscitation ALDH2 activity was significantly decreased and its expression was markedly reduced in the kidney and intestine in those resuscitated animals compared with the sham group; nevertheless, both of them were significantly greater in those animals receiving Alda-1 treatment compared to the CPR group. In addition, renal, intestinal apoptosis and ferroptosis after resuscitation were observed in the CPR and CPR+Alda-1 groups, in which both of them were significantly milder in the CPR+Alda1 group than in the CPR group.

**Conclusions:**

The activation of ALDH2 by Alda-1 treatment significantly alleviated post-resuscitation renal and intestinal injuries through the inhibition of cell apoptosis and ferroptosis in a pig model of CA and resuscitation.

## Introduction

Cardiac arrest (CA) remains a high incidence rate accompanied by high rates of morbidity and mortality in the world (1–3). It has been reported that the incidence of out-of-hospital CA was 88.8 individuals per 1,000,000 population in United States in 2020, in which the survival rates of hospital admission and discharge were 24.0 and 9.0%, respectively (1). After those CA victims initially obtain successful cardiopulmonary resuscitation (CPR), the ensuing myocardial and neurological dysfunction is considered as the main contributors to their poor outcomes (4, 5). However, growing evidence shows that almost 50% of CA survivors will develop acute kidney injury, which can cause the accumulation of toxic metabolites and the occurrence of electrolyte disturbance, and further might result in poor neurological outcomes and even death (6–8). In addition, the intestine is sensitive to systemic ischemia/reperfusion (IR) injury following CA and resuscitation, and its barrier dysfunction may promote bacterial translocation into the blood and finally the occurrence of sepsis and multiple organ dysfunction (9, 10). Thus, it is needed to explore the feasible strategies for the treatment of renal and intestinal injuries after CA and resuscitation.

Recently, Alda-1, a specific activator of aldehyde dehydrogenase 2 (ALDH2), has been shown to protect several target organs against regional IR injury through enhancing ALDH2-mediated detoxification of reactive aldehydes (11). Especially, two studies have manifested that the activation of ALDH2 by Alda-1 treatment is beneficial to mitigate renal and intestinal injuries in those regional IR models (12, 13). In the setting of CA and resuscitation, another two studies have also confirmed that Alda-1 treatment is an effective therapeutic intervention for improving post-resuscitation myocardial dysfunction (14, 15). However, it is unclear whether Alda-1 can protect the kidney and intestine against systemic IR injury triggered by CA and resuscitation.

Currently, we employed a clinically relevant pig model, and investigated the effects of Alda-1 treatment on renal and intestinal injuries after CA and resuscitation. In addition, considering that the classical and novel forms of programmed cell death, cell apoptosis and ferroptosis are both involved in the pathogenesis of regional renal and intestinal IR injury (16, 17), their potential regulation by Alda-1 treatment was also explored in the present model of systemic IR injury. We hypothesized that the activation of ALDH2 by Alda-1 treatment would alleviate post-resuscitation renal and intestinal injuries in this pig model of CA and resuscitation, in which the protective role of Alda-1 was related to the inhibition of cell apoptosis and ferroptosis.

## Materials and Methods

All animals received humane care in compliance with the Institutional Animal Care and Use Committee guidelines. Twenty-four healthy male white domestic pigs, aged 4–6 months, weighing 39 ± 2 kg, were supplied by Shanghai Jiagan Biotechnology Inc. (Shanghai, China). The animals were fed under the conditions of standard atmospheric pressure, 12/12 h light/dark cycle, room temperature (20–25°C), humidity (60–80%), closed cage, spontaneous water intake, regular feeding, regular cleaning, and disinfection. This study was approved by the Institutional Animal Care and Use Committee of the First People's Hospital of Fuyang Hangzhou, Zhejiang Chinese Medical University.

### Animal Preparation

The animals were fasted for 12 h before the experiment. Thereafter, they were anesthetized by an intramuscular injection of midazolam (0.4–0.5 mg/kg) and then an intravenous injection of propofol (2 mg/kg) followed by the maintenance of continuous infusion of propofol (4 mg/kg/h). For pain medication, a dose of 6 μg/kg of fentanyl was intramuscularly injected every 6 h. In addition, normal saline (5 ml/kg/h) was continuously infused to maintain fluid balance. Subsequently, they were intubated with a cuffed endotracheal tube and then ventilated with an emergency and transport ventilator (Oxylog 3000 plus, Drager, Luebeck, Germany), in which the ventilator setting was 10 ml/kg of tidal volume, 12 bpm of respiratory rate, 40 L/min of peak flow and 21% of oxygen concentration. In addition, end-tidal CO_2_ was monitored by an M Series Monitor Defibrillator (ZOLL Medical Corporation, Chelmsford, MA), and the electrocardiogram was recorded by a patient monitoring system (BeneVision N22, Mindray, Shenzhen, China).

For the measurement of aortic pressure, one fluid-filled 7 Fr thermodilution-tipped catheter was advanced from the right femoral artery into the thoracic aorta. For the measurement of right atrial pressure and the collection of blood samples, another fluid-filled 7 Fr thermodilution-tipped catheter was advanced from the right femoral vein into the right atrium. The data of aortic and right atrial pressures were recorded by the patient monitoring system. All catheters were intermittently flushed with normal saline containing 5 IU bovine heparin per ml. For the induction of CA, a 5 F pacing catheter was advanced from the right external jugular vein into the right ventricle. Rectal temperature was monitored and maintained at a normal level of 38.0 ± 0.5°C with the aid of the Blanketrol III Hyper-Hypothermia System (Cincinnati Sub-Zero, Cincinnati, OH) throughout the experiment.

### Experimental Procedures

After animal preparation was finished and baseline measurements were obtained, the animals were then randomly divided into one of the three groups: sham (*n* = 6), CPR (*n* = 10), CPR+Alda-1 (*n* = 8). In the CPR+Alda-1 group, a dose of 0.88 mg/kg of Alda-1 (Selleck Chemicals LLC., Houston, TX) was intravenously administered at 5 min after successful CPR. This dose of Alda-1 was chosen based on the previous studies and the principle of dose conversion (13, 18, 19), in which Alda-1 treatment was shown to effectively alleviate regional renal and intestinal IR injury in those small-animal models. In the other two groups, the same volume of vehicles was intravenously infused at the same time point.

Sham animals underwent surgical preparation only. In the CPR and CPR+Alda-1 groups, the animal model was established by 8 min of CA and then 8 min of CPR. First, CA was electrically induced by delivering a 1 mA alternating current to the right ventricular endocardium. Once CA was confirmed, mechanical ventilation was discontinued and the pacing catheter was withdrawn. Second, CPR was performed by continuous chest compression and mechanical ventilation after 8 min of untreated CA. Chest compression was provided by two professional CPR providers, in which its quality was monitored using a CPR feedback device (PalmCPR, Sunlife, Shanghai, China) to maintain a compression depth of 50–60 mm at a rate of 100–120 per min. The working parameters of the ventilator were set with 7 ml/kg of tidal volume, 10 bpm of respiratory rate, and 100% of oxygen concentration. A dose of 20 μg/kg of epinephrine was administered at 2 min of CPR, followed by the same administration at an interval of 4 min. Third, a 150-J biphasic electrical shock was delivered by the M Series Monitor Defibrillator at 8 min of CPR. If an organized cardiac rhythm was restored and a mean arterial pressure of more than 50 mmHg was achieved for 5 min or more, the animal was considered as successful CPR. If not, CPR was immediately resumed for 2 min before another electrical shock. This protocol was continued until successful CPR or for a total of 18 min. Following successful CPR, mechanical ventilation was continued with the same setting before CA, and meanwhile, the anesthesia was maintained for 4 h. Subsequently, the catheters were removed, the wounds were sutured, and the endotracheal tube was removed for the animals. After that, they were returned to observe for an additional 20 h in their cages. During observation, the animals had free access to water and food, and the dose of 1.0 g of cefotiam was intramuscularly injected at 4, and 16 h post-resuscitation to prevent the infection. Finally, the animals were euthanized with an intravenous injection of propofol (3 mg/kg) and then potassium chloride (10 mol/L, 10 ml). The necropsy was routinely performed to record those possible injuries resulting from the surgical or CPR intervention or the presence of obfuscating diseases.

### Measurements

Renal and intestinal injury biomarkers including creatinine (Cr), blood urea nitrogen (BUN), intestinal fatty acid-binding protein (IFABP), and diamine oxidase (DAO) were measured at baseline, 1, 2, 4, and 24 h after resuscitation. First, venous blood samples were collected at these time points. Subsequently, the serums were obtained by centrifugation at 2,000 rpm for 20 min. Thereafter, the levels of biomarkers of renal and intestinal injuries were measured with enzyme-linked immunosorbent assay kits (Meixuan Biotechnology Inc., Shanghai, China) according to the manufacturer's instructions.

For the evaluation of ALDH2 activity in the kidney and intestine, left kidney and distal ileum were harvested immediately after the animals were euthanized at 24 h post-resuscitation. Subsequently, tissue specimens were homogenized with the iced saline, and then the supernatants were obtained by centrifugation at 4,000 rpm at 4°C for 15 min. Thereafter, ALDH2 activity was detected with the mitochondrial ALDH2 activity assay kit (Abcam, Cambridge, UK) according to the manufacturer's instructions. For the evaluation of oxidative stress in the kidney and intestine, the supernatants were similarly obtained from tissue specimens to detect the contents of malondialdehyde (MDA), 4-hydroxy-2-nonenal (4-HNE), and glutathione (GSH) with their assay kits according to the manufacturer's instructions. The assay kits of MDA and GSH were purchased from Nanjing Jiancheng Bioengineering Institute (Nanjing, China), and the assay kit of 4-HNE was purchased from Abcam plc. (Cambridge, UK).

For the measurement of cell apoptosis in the kidney and intestine, tissue specimens harvested from left kidney and distal ileum at 24 h post-resuscitation were fixed in 4% paraformaldehyde for 24 h, then embedded in paraffin, and finally cut in a 5-μm section. Subsequently, the sections were stained with the TdT-mediated dUTP nick end labeling (TUNEL) assay kit (Boster Biological Technology co, Wuhan, China) according to the manufacturer's instructions. After that, six views were randomly captured at ×200 magnification under an optical microscope (Biological microscope C × 31, Olympus, Japan), then the numbers of TUNEL-positive cells and total cells in each view were counted, and finally, their ratio was considered as the rate of apoptotic cells. The level of iron deposition in the kidney and intestine was measured by Prussian blue staining. After the sections were obtained according to the methods mentioned above, they were then rehydrated and incubated with Prussian blue staining solution, thereafter rinsed in distilled water and stained with nuclear fast red, and finally photographed at ×200 magnification under an optical microscope. The percentage of positive staining area was analyzed and calculated.

For the evaluation of expression levels of ALDH2, and ferroptosis-related proteins acyl-CoA synthetase long-chain family member 4 (ACSL4) and glutathione peroxidase 4 (GPX4) in the kidney and intestine, tissue specimens were similarly harvested at 24 h post-resuscitation. Subsequently, tissue protein was extracted with the RIPA lysate, then centrifuged at 12,000 rpm at 4°C for 20 min, and finally quantified by a BCA protein quantitation kit (Beyotime Biotechnology, Shanghai, China). After that, the protein sample was separated by 10% SDS-PAGE, transferred to a PVDF membrane, and blocked in 5% non-fat milk. All membranes were incubated with primary anti-ALDH2 (1:1,000, Proteintech, Rosemount, IL), anti-ACSL4 (1:1,000, Proteintech, Rosemount, IL), anti-GPX4 (1:1,000, Proteintech, Rosemount, IL), and anti-glyceraldehyde-3-phosphate dehydrogenase (GAPDH) (1:1,000, BBI Life Science Corporation, Shanghai, China) at 4°C for 24 h, and then incubated with the secondary antibody (1:5,000, BBI Life Science Corporation, Shanghai, China) at room temperature for 1 h. The protein was visualized with ECL luminescent substrates and analyzed by Image J software (NIH, Bethesda, United States).

### Statistical Analysis

Continuous variables were presented as mean ± SD when the data were confirmed to be normally distributed or as a median (25th, 75th percentiles) when the data were not normally distributed. The distribution of continuous variables was analyzed using the Kolmogorov-Smirnov test. Subsequently, the comparisons between two groups were performed by Student's *t*-test, among more than two groups by one-way analysis of variance or the Kruskal-Wallis test for non-parametric data. Comparisons between time-based measurements within each group were performed with repeated-measurement analysis of variance. When a significant difference was observed in the overall comparison of groups, the comparisons between any other two groups were further analyzed by the Bonferroni test. For the comparison of categorical variables such as the rates of resuscitation success and 24-h survival, Fisher's exact test was used. A value of *P* < 0.05 was considered significant.

## Results

**Figure 1:**
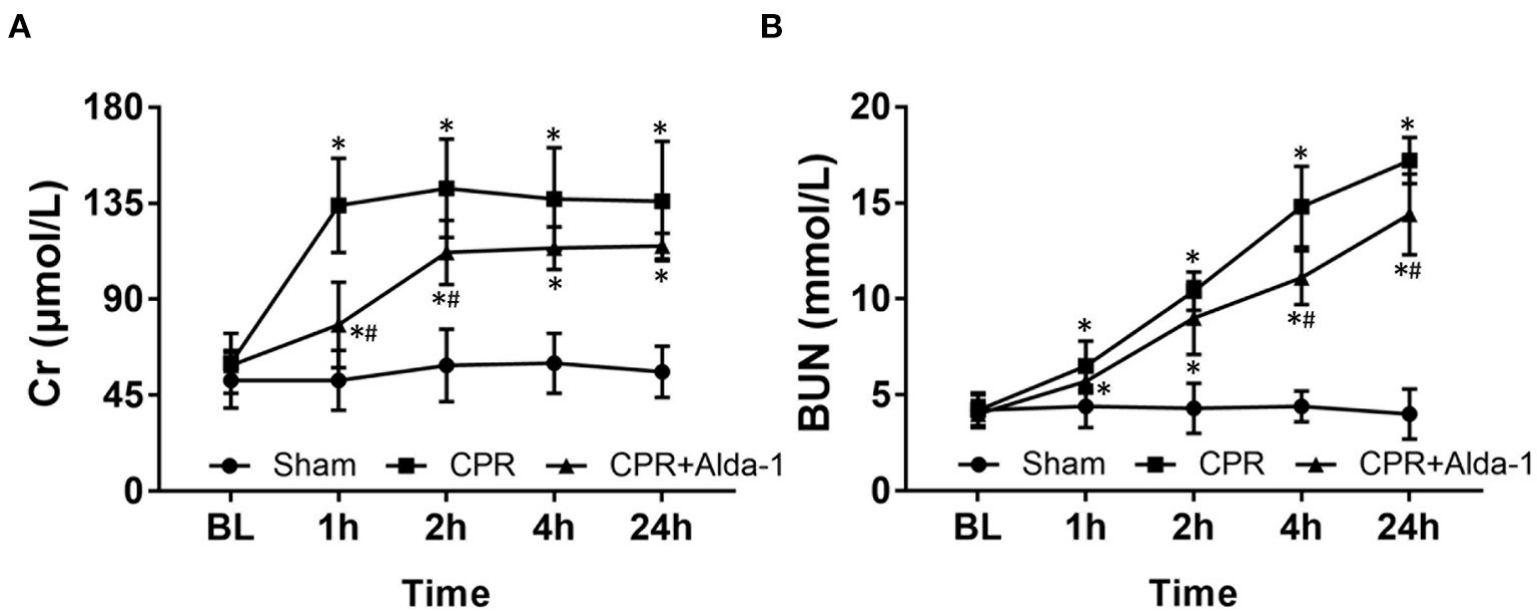
The changes of serum creatinine (Cr) and blood urea nitrogen (BUN) in the three groups. **(A)** Cr. **(B)** BUN. BL, baseline; CPR, cardiopulmonary resuscitation. The sham group contained 6 pigs throughout the experiment. The CPR group had 10 pigs at baseline, 5 pigs at 1, 2, 4, and 24 h after resuscitation. The CPR+Alda-1 group had 8 pigs at baseline, and 6 pigs at 1, 2, 4, and 24 h after resuscitation. *P < 0.05 vs. Sham group; ^#^*P* < 0.05 vs. CPR group.

**Figure 2:**
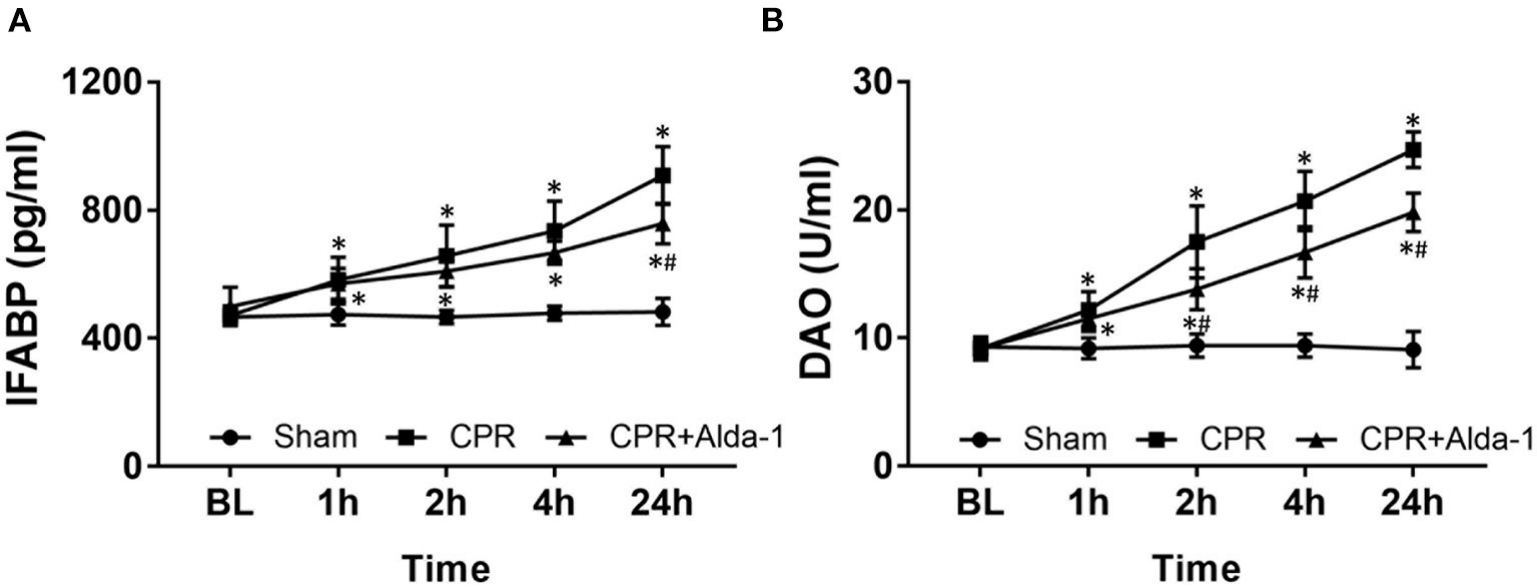
The changes of serum intestinal fatty acid binding protein (IFABP) and diamine oxidase (DAO) in the three groups. **(A)** IFABP. **(B)** DAO. BL, baseline; CPR, cardiopulmonary resuscitation. The sham group contained 6 pigs throughout the experiment. The CPR group had 10 pigs at baseline, 5 pigs at 1, 2, 4, and 24 h after resuscitation. The CPR+Alda-1 group had 8 pigs at baseline, and 6 pigs at 1, 2, 4, and 24 h after resuscitation. *P < 0.05 vs. Sham group; ^#^*P* < 0.05 vs. CPR group.

A total of 24 pigs were utilized in this study. There were no differences in baseline renal and intestinal injury biomarkers including Cr, BUN, IFABP, and DAO among the three groups (Figures 1, 2). Subsequently, the same procedure of CA and CPR was performed in the CPR and CPR+Alda-1 groups. Consequently, five of the 10 animals in the CPR group and six of the eight animals in the CPR+Alda-1 group were successfully resuscitated, and thereafter all those resuscitated animals survived for 24 h; in which the rates of resuscitation success and 24-h survival were not significantly different between the two groups (both *P* = 0.367).

After resuscitation, the levels of Cr and BUN in serum were significantly increased at all time points in those animals experiencing CA and resuscitation when compared with the sham group. However, the increases in Cr and BUN were slower in those animals treated with Alda-1, in which the serum levels of Cr at 1, and 2 h post-resuscitation and the serum levels of BUN at 4, and 24 h post-resuscitation were significantly decreased when compared to the CPR group (Figure 1).

After resuscitation, the serum levels of IFABP and DAO indicating the severity of intestinal mucosal injury were significantly greater at all time points in those resuscitated animals when compared with the sham group. However, both of them were always lower in the CPR+Alda-1 group than in the CPR group, in which the serum levels of IFABP at 24 h post-resuscitation and the serum levels of DAO starting 2 h after resuscitation were significantly different between the two groups (Figure 2).

**Figure 3:**
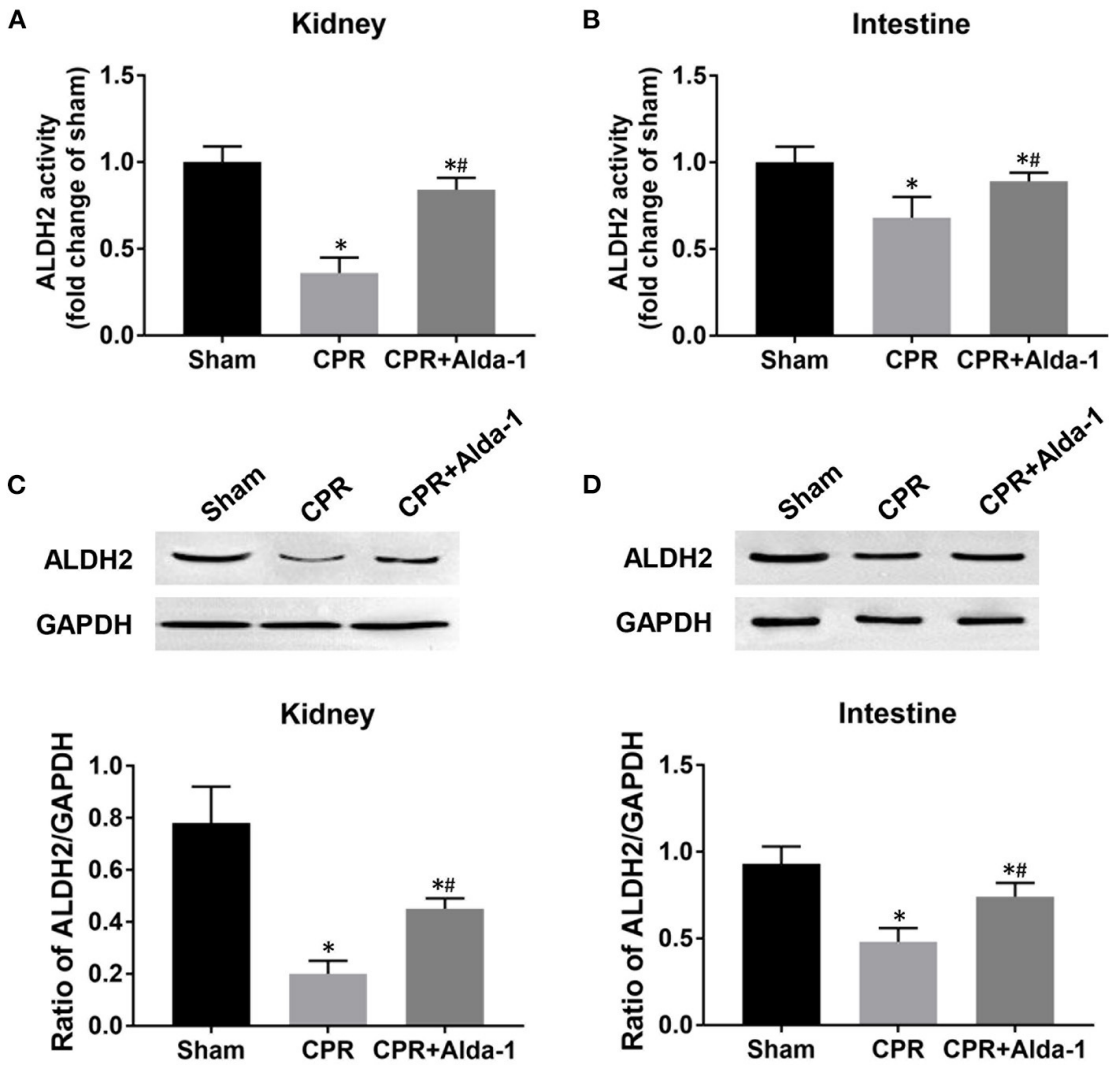
The changes of aldehyde dehydrogenase 2 (ALDH2) activity and expression in the kidney and intestine in the three groups. **(A)** Renal ALDH2 activity. **(B)** Intestinal ALDH2 activity. **(C)** Renal ALDH2 expression. **(D)** Intestinal ALDH2 expression. CPR, cardiopulmonary resuscitation; GAPDH, glyceraldehyde-3-phosphate dehydrogenase. Tissue samples were measured at 24 h after resuscitation, in which the sham, CPR, and CPR+Alda-1 groups contained 5 pigs, respectively. *P < 0.05 vs. Sham group; ^#^*P* < 0.05 vs. CPR group.

At 24 h post-resuscitation, the activities of ALDH2 in the kidney and intestine were significantly decreased in the CPR and CPR+Alda-1 groups when compared with the sham group. However, renal and intestinal ALDH2 activities were partly restored in those animals treated with Alda-1, and its values were significantly greater than those in the CPR group (Figures 3A,B). Likewise, the expression levels of ALDH2 in the kidney and intestine were significantly reduced in the CPR and CPR+Alda-1 groups when compared with the sham group. Nevertheless, Alda-1 treatment significantly increased renal and intestinal ALDH2 expression when compared to the CPR group (Figures 3C,D).

**Figure 4:**
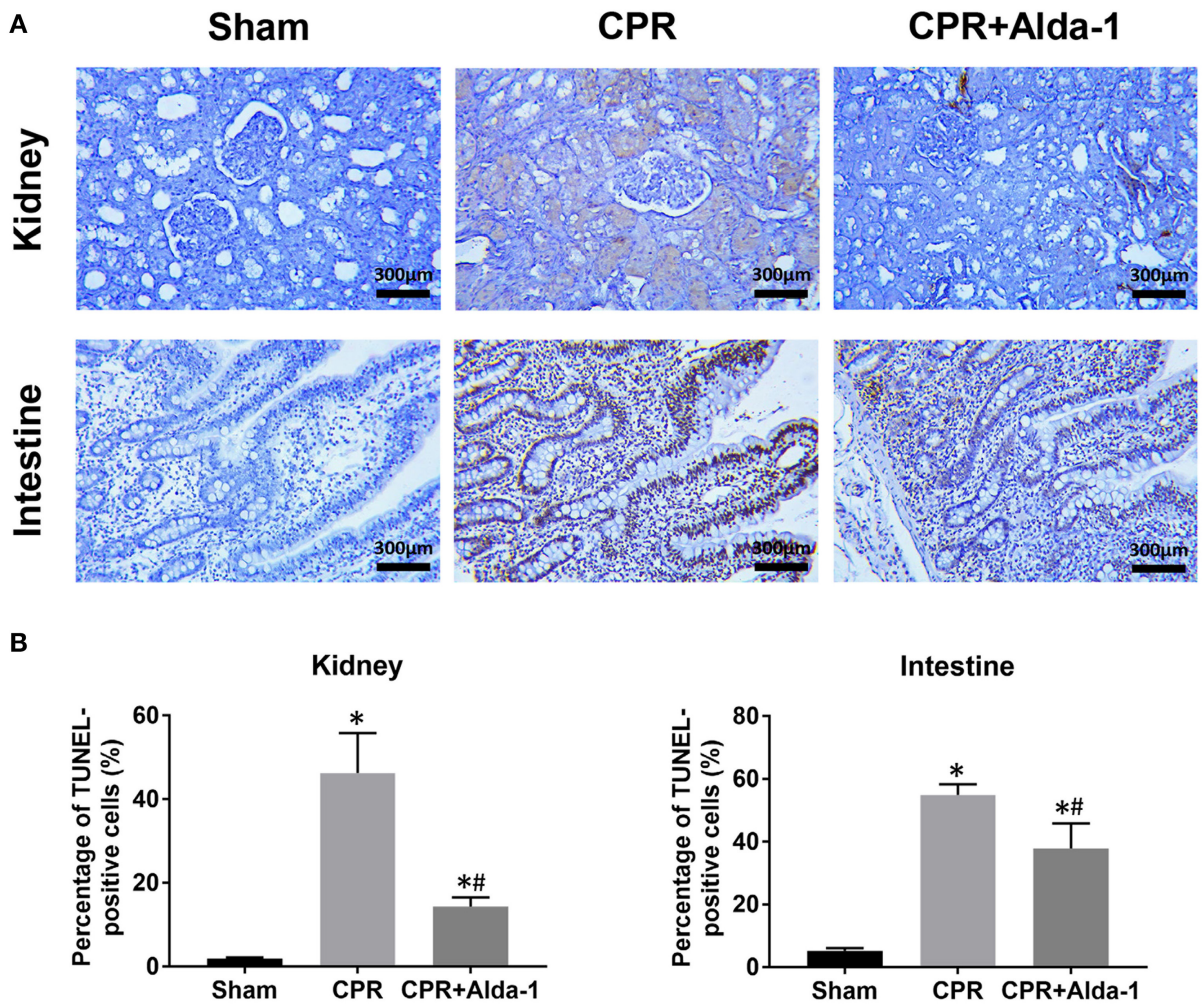
The changes of cell apoptosis in the kidney and intestine in the three groups. **(A)** Representative photomicrographs of TdT-mediated dUTP nick end labeling (TUNEL) assay. **(B)** The percentage of TUNEL-positive cells. CPR, cardiopulmonary resuscitation. Tissue samples were measured at 24 h after resuscitation, in which the sham, CPR, and CPR+Alda-1 groups contained 5 pigs, respectively. *P < 0.05 vs. Sham group; ^#^*P* < 0.05 vs. CPR group.

At 24 h post-resuscitation, the percentage of TUNEL-positive cells was significantly increased in the kidney and intestine in the CPR and CPR+Alda-1 groups when compared with the sham group. However, the percentage of TUNEL-positive cells was significantly lower in those animals receiving Alda-1 treatment, which indicated that renal and intestinal apoptosis was significantly alleviated in the CPR+Alda-1 group when compared to the CPR group (Figure 4).

**Figure 5:**
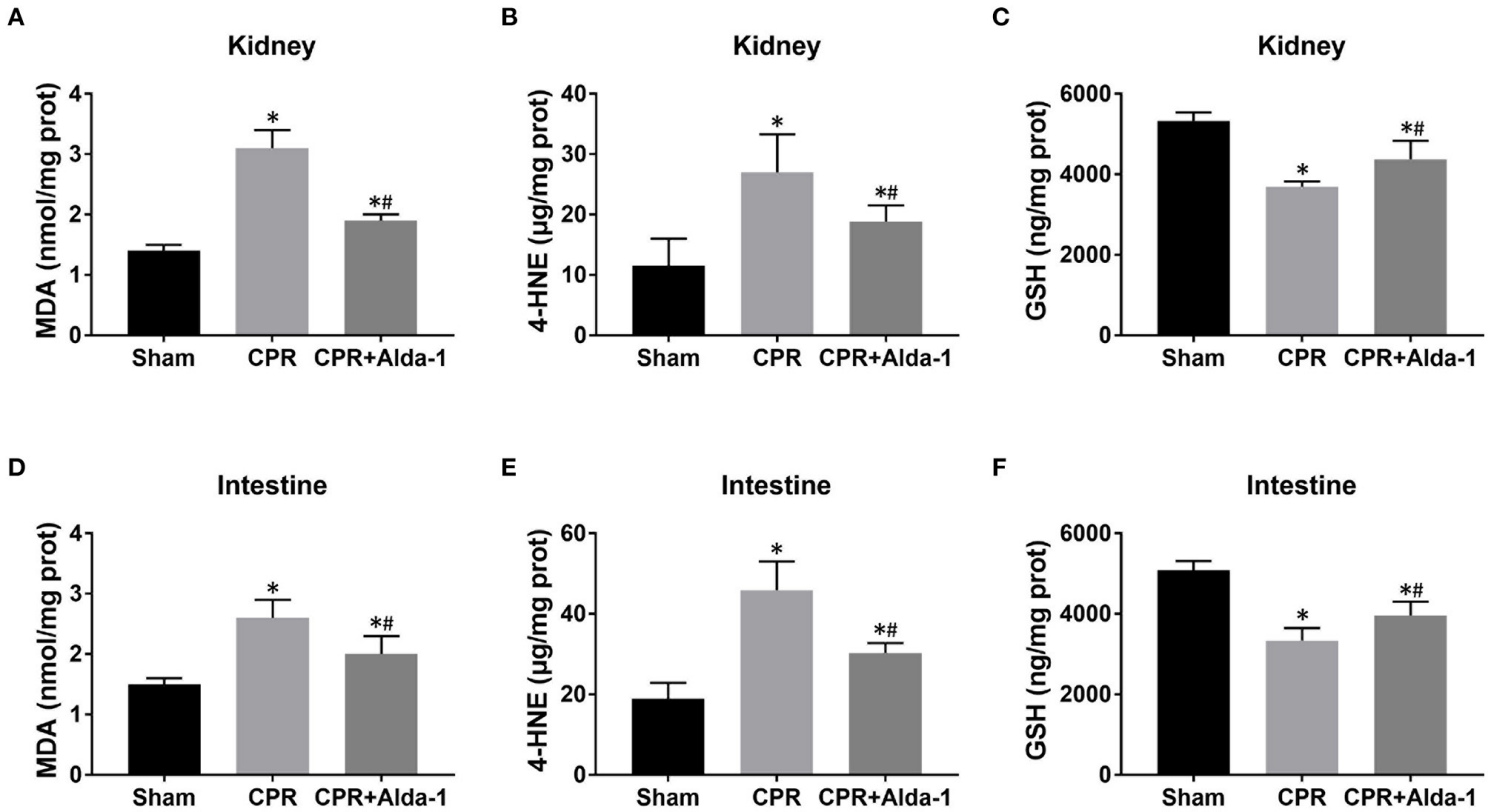
The changes of oxidative stress in the kidney and intestine in the three groups. **(A–C)** Renal malondialdehyde (MDA), 4-hydroxy-2-nonenal (4-HNE), and glutathione (GSH) contents. **(D–F)** Intestinal MDA, 4-HNE, and GSH contents. CPR, cardiopulmonary resuscitation. Tissue samples were measured at 24 h after resuscitation, in which the sham, CPR, and CPR+Alda-1 groups contained 5 pigs, respectively. *P < 0.05 vs. Sham group; ^#^*P* < 0.05 vs. CPR group.

**Figure 6:**
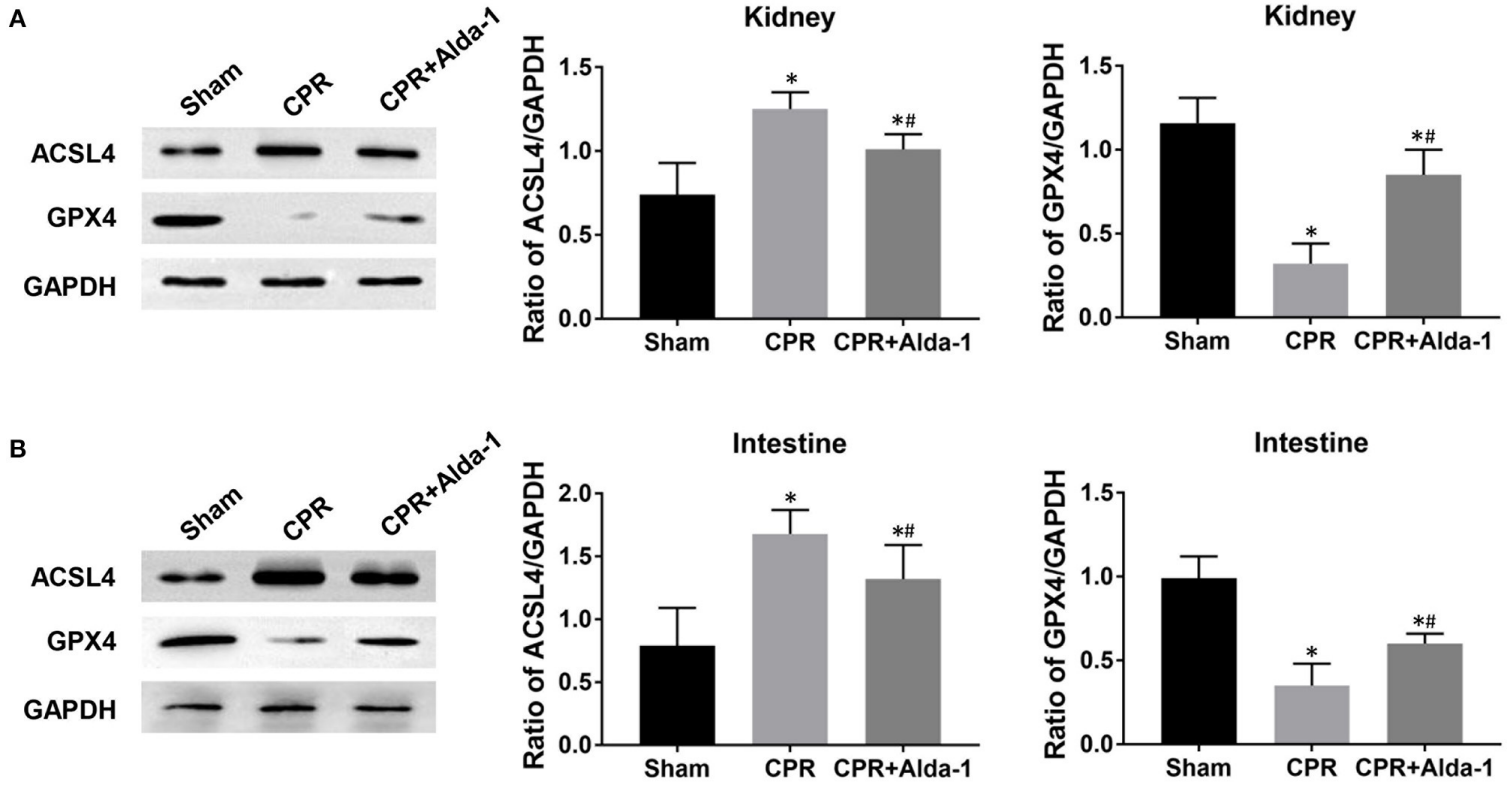
The changes of ferroptosis-related proteins in the kidney and intestine in the three groups. **(A)** The expression levels of ACSL4 (acyl-CoA synthetase long-chain family member 4) and glutathione peroxidase 4 (GPX4) in the kidney. **(B)** The expression levels of ACSL4 and GPX4 in the intestine. CPR, cardiopulmonary resuscitation. Tissue samples were measured at 24 h after resuscitation, in which the sham, CPR, and CPR+Alda-1 groups contained 5 pigs, respectively. *P < 0.05 vs. Sham group; ^#^*P* < 0.05 vs. CPR group.

**Figure 7:**
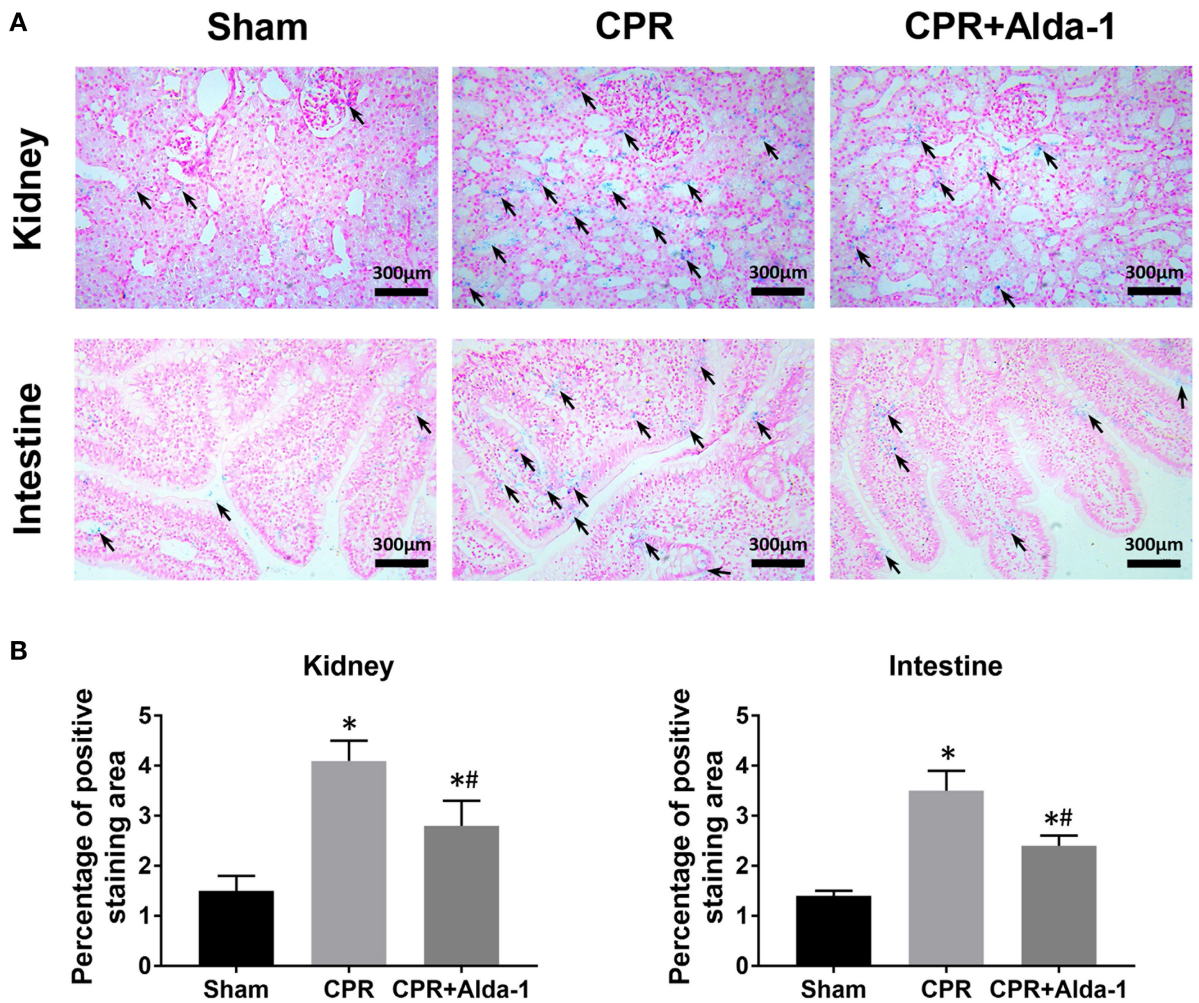
The changes of iron deposition in the kidney and intestine in the three groups. **(A)** Representative photomicrographs of Prussian blue staining (200× magnification); **(B)** The percentage of positive staining area. CPR, cardiopulmonary resuscitation. Tissue samples were measured at 24 h after resuscitation, in which the sham, CPR, and CPR+Alda-1 groups contained 5 pigs, respectively. *P < 0.05 vs. Sham group; ^#^*P* < 0.05 vs. CPR group.

To investigate the effects of Alda-1 on renal and intestinal ferroptosis after CA/CPR in pigs, we examined the changes of iron overload, lipid peroxidation product, antioxidant and ferroptosis-related proteins in the kidney and intestine at 24 h after resuscitation. Consequently, iron deposition, MDA and 4-HNE contents, ACSL4 expression were significantly increased while GSH content and GPX4 expression were significantly decreased in the kidney and intestine in the CPR and CPR+Alda-1 groups when compared with the sham group. However, all of these changes were significantly milder in the CPR+Alda-1 group than in the CPR group, which indicated that Alda-1 treatment significantly alleviated renal and intestinal ferroptosis when compared to the CPR group (Figures 5–7).

## Discussion

The present study employed a clinically relevant, large-animal model, and demonstrated that the activator of ALDH2, Alda-1 administered after successful CPR effectively alleviated post-resuscitation renal and intestinal injuries when compared with the CPR group. Additionally, Alda-1 treatment produced post-resuscitation renal and intestinal protection possibly through the inhibition of cell apoptosis and ferroptosis.

ALDH2 is one kind of mitochondrial enzyme expressed in a variety of organs, which plays an important role in the clearance of toxic aldehydic products through its oxidation under various stress conditions (11). In the setting of regional IR injury, the activation of ALDH2 by its specific agonist Alda-1 has been confirmed to be an effective approach for organ protection. In 2013, Fu et al. (20) demonstrated that the activation of ALDH2 by Alda-1 treatment decreased the accumulation of reactive aldehydes after cerebral IR injury and therefore improved brain infarct volume and neurological function. In 2016, ding et al. (21) demonstrated that Alda-1 pre-treatment attenuated lung IR injury possibly through the activation of ALDH2 to remove 4-HNE in pulmonary alveolar epithelial cells. The same year, Ji et al. (22) demonstrated that Alda-1 treatment protected the heart against IR injury by suppressing mitophagy through reducing reactive oxygen species production. In 2018, Li et al. (23) demonstrated that Alda-1 treatment alleviated liver IR injury through direct clearance of reactive aldehydes and indirect enhancement of autophagy *via* AMPK activation. Recently, one study demonstrated for the first time that Alda-1 treatment alleviated intestinal IR injury through the inhibition of oxidative stress, inflammatory response, and cell apoptosis (13). Two other studies demonstrated that ALDH2 overexpression mitigated IR-induced acute kidney injury by regulating cell autophagy *via* the beclin-1 pathway, and its activation by Alda-1 treatment alleviated renal IR injury in hypothermic machine perfusion through the Akt-mTOR autophagy signaling pathway (12, 18).

In the setting of systemic IR injury, two studies have explored the protective role of Alda1 in post-resuscitation myocardial dysfunction, in which cardiac outcome was improved through suppressing mitochondrial reactive oxygen species production and restoring calcium/calmodulin-dependent protein kinase II homeostasis, respectively (14, 15). However, no investigation has reported the potential effects of Alda-1 treatment on renal and intestinal injuries after CA and resuscitation. In the present study, significantly decreased ALDH2 activity and expression in the kidney and intestine was observed in those animals experiencing CA and resuscitation; however, both of them were markedly restored in animals treated with Alda-1 when compared with the CPR group. Accordingly, the activation of ALDH2 by Alda-1 treatment significantly reduced the levels of biomarkers of renal and intestinal injuries accompanied with significantly milder renal and intestinal apoptosis when compared to the CPR group. Thus, Alda-1 treatment could become an effective approach for renal and intestinal protection after CA and resuscitation.

Ferroptosis is a newly discovered, non-apoptotic form of regulated cell death driven by iron overload and lipid peroxidation, which has been considered as the key factor leading to IR injury and organ failure (24). In the setting of renal IR injury, Su et al. (16) demonstrated that Pannexin 1 deletion protected against renal IR injury by targeting ferroptotic cell death *via* the mitogen-activated protein kinase/extracellular signal-regulated kinase pathway. Zhao et al. (25) demonstrated that XJB-5-131 attenuated IR-induced renal injury and inflammation by specifically inhibiting tubular epithelial ferroptosis rather than necroptosis and pyroptosis. In the setting of intestinal IR injury, Deng et al. (17) demonstrated that capsiate alleviated ferroptosis-dependent intestinal I/R injury by enhancing GPX4 expression through activating transient receptor potential vanilloid 1. Li et al. (26) demonstrated that ACSL4 expression was promoted by special protein 1 after intestinal I/R injury, and its inhibition protected against ferroptotic cell death and thereby alleviated intestinal tissue injury. Currently, almost no investigation has reported the phenomenon of cell ferroptosis in the kidney and intestine after CA and resuscitation. In addition, it is unknown whether cell ferroptosis is a potential target by which Alda-1 alleviates IR-induced organ injury. However, a recent study demonstrated for the first time that Alda-1 could rescue cardiac contractile dysfunction induced by Alzheimer's disease through the inhibition of ACSL4-dependent ferroptosis (27). In the present study, significantly increased iron deposition, MDA and 4-HNE contents, and ACSL4 expression, and meanwhile significantly decreased GSH content and GPX4 expression were observed in the kidney and intestine in those resuscitated animals, which indicated the occurrence of cell ferroptosis in these two organs after CA and resuscitation. Nevertheless, Alda-1 treatment significantly reversed these changes mentioned above when compared with the CPR group. Thus, Alda-1 treatment could alleviate post-resuscitation renal and intestinal injuries through the inhibition of cell ferroptosis.

There were some limitations in this study. First, only one single dose of Alda-1 was administered after successful CPR to investigate its effectiveness in this pig study. Thus, its feasible dose range and therapeutic time window need to be further confirmed. Second, a shorter duration of 24 h of observation was set to evaluate the protective role of Alda-1 in the present study. Considering that renal and intestinal injuries were still severe at the end of the experiment, a longer observation period is needed to fully confirm the protective effects produced by Alda-1 treatment in the future. Third, although cell apoptosis and ferroptosis observed in the kidney and intestine after resuscitation was successfully inhibited by Alda-1 treatment; however, the potential protective mechanism requires further investigation.

## Conclusions

In a pig model of CA and resuscitation, the activation of ALDH2 by Alda-1 treatment significantly alleviated post-resuscitation renal and intestinal injuries, in which the protective effects were related to the inhibition of cell apoptosis and ferroptosis.

## Data Availability Statement

The original contributions presented in the study are included in the article/supplementary materials, further inquiries can be directed to the corresponding author.

## Ethics Statement

The animal study was reviewed and approved by the Institutional Animal Care and Use Committee of the First People's Hospital of Fuyang Hangzhou, Zhejiang Chinese Medical University.

## Author Contributions

QY and JG designed the study and performed the measurements. QY, JG, XS, and LC performed the experiments. QY, JG, and WL provided the materials. XS, LC, and LJ analyzed the data. QY wrote the manuscript. All authors contributed to the article and approved the submitted version.

## Funding

This study was supported by the Hangzhou Medical Science Foundation of Zhejiang Province of China (2018B053), the Fuyang Medical Science Foundation of Hangzhou of Zhejiang Province of China (2019SK006), and the Hangzhou Scientific Research Project of Zhejiang Province of China (20181228Y145 and 20211231Y171).

## Conflict of Interest

The authors declare that the research was conducted in the absence of any commercial or financial relationships that could be construed as a potential conflict of interest.

## Publisher's Note

All claims expressed in this article are solely those of the authors and do not necessarily represent those of their affiliated organizations, or those of the publisher, the editors and the reviewers. Any product that may be evaluated in this article, or claim that may be made by its manufacturer, is not guaranteed or endorsed by the publisher.
